# The Replicative Consequences of Papillomavirus E2 Protein Binding to the Origin Replication Factor ORC2

**DOI:** 10.1371/journal.ppat.1005934

**Published:** 2016-10-04

**Authors:** Marsha DeSmet, Sriramana Kanginakudru, Anne Rietz, Wai-Hong Wu, Richard Roden, Elliot J. Androphy

**Affiliations:** 1 Department of Dermatology, Indiana University School of Medicine, Indianapolis, Indiana, United States of America; 2 Department of Pathology, Johns Hopkins University, Baltimore, Maryland, United States of America; 3 Department of Microbiology and Immunology, Indiana University School of Medicine, Indianapolis, Indiana, United States of America; Penn State University School of Medicine, UNITED STATES

## Abstract

The origin recognition complex (ORC) coordinates a series of events that lead to initiation of DNA strand duplication. As a nuclear double stranded DNA plasmid, the papillomavirus (PV) genome resembles a mini-chromosome in infected cells. To initiate its replication, the viral E2 protein binds to and recruits the E1 DNA helicase at the viral origin. PV genome replication program exhibits three stages: initial amplification from a single genome upon infection to a few copies per cell, a cell cycle linked maintenance phase, and a differentiation dependent late stage where the genome is amplified to thousands of copies. Involvement of ORC or other pre-replication complex (pre-RC) factors has not been described. We report that human PV (HPV) and bovine PV (BPV-1) E2 proteins bind to ORC2, however, ORC2 was not detected at the viral origin. Depletion of ORC2 enhanced PV replication in a transient replication model and in keratinocytes stably maintaining viral episomes, while there was no effect on copy number in a cell line with integrated HPV genomes. Consistent with this, occupancy of E1 and E2 at the viral origin increased following ORC2 silencing. These data imply that ORC2 is not necessary for activation of the PV origin by E1 and E2 but instead suppresses E2 replicative function. Furthermore, we observed that over-expression of HPV E2 decreased ORC2 occupation at two known mammalian origins of replication, suggesting that E2 restricts pre-ORC assembly that could otherwise compete for host replication complexes necessary for viral genome amplification. We infer that the ORC2 complex with E2 restricts viral replication in the maintenance phase of the viral replication program and that elevated levels of E2 that occur during the differentiation dependent amplification stage subvert ORC loading and hence DNA synthesis at cellular origins.

## Author Summary

Papillomavirus genome replication occurs during three distinct stages that are linked to the differentiation state of the infected epithelium. The viral proteins E1 and E2 recognize the viral origin and initiate a process that attracts host DNA replication factors. The origin recognition complex (ORC) coordinates initiation of chromosome duplication. While ORC2 binds to the E2 protein, its depletion does not impair PV genome replication. Instead, depletion of ORC2 stimulates viral replication, while over-expression of E2 protein decreases ORC2 occupancy at mammalian origins. We propose that the relative abundance of E2 and ORC2 in complex regulates viral and cellular origin licensing.

## Introduction

Papillomaviruses (PV) are medically important pathogens especially as specific genotypes carry a high risk of progression to cancer, most commonly of the uterine cervix and oropharynx. Because PVs have limited protein coding capacity in their typically 8 kilobases (kb) genome, these viruses do not encode a DNA polymerase and must rely on host DNA replication factors. The viral genome replicates and is maintained as circular covalently closed double stranded, histone coated DNA plasmids in infected cells, thus resembling multi-copy mini-chromosomes. The viral genome replicative program consists of three stages [1, 2]. Upon virus infection, its genome enters the nucleus of basal level epithelial cells and establishes a low copy number (1 to perhaps 50). In the second ‘maintenance’ stage, these episomes duplicate as host epithelial cells replicate and depart the basal cell and suprabasal compartments [3, 4]. Monolayer keratinocyte cultures that harbor viral episomes reflect this stage of virus replication. During this stage, the autonomous viral genomes segregate in mitosis as a kinetochore independent mini-chromosome. E2 protein binding to ChlR1 and Brd4 was shown to mediate attachment of the viral DNA to host chromosomes that is necessary for mitotic partitioning and nuclear retention of viral episomes [5, 6]. The third ‘amplification’ stage occurs in upper epithelial strata where non-dividing epithelial cells persist in a prolonged S/G2 phase [7]. In these cells, the viral episomes replicate to hundreds of episomes that are packaged into nascent virion particles.

Many of our insights into PV replication proteins emerged from studies of bovine papillomavirus type-1 (BPV), which is maintained as a stable replicating episome in murine NIH3T3 and C127 cell lines. Its E2 protein is composed of an N-terminal 220 amino acid transactivation domain (TAD), a non-conserved hinge region, and a C-terminal dimerization and DNA binding domain [8]. The TAD mediates interactions with several cellular proteins necessary for transcriptional activation and replication such as Brd4, TaxBP1, and GPS2/AMF-1 [6, 9–11]. The E2 protein binds with high affinity to an inverted palindromic sequences present in all PVs, which serves to regulate viral transcription and replication [12]. E2 binds to and recruits E1, an ATP dependent replicative helicase, to these E2-binding motifs [13]. Together with an adjacent E1 binding site and short polyA tract, these composite sequences define and function as the origin of replication (ori) [13, 14]. Antibodies to BPV E1 and E2 have been used in ChIP experiments to document localization of E2 protein to this region in G1/S phase [15], however, analogous reagents for the HPV protein counterparts have not been available. Assembly of E1 into double hexamers requires release from E2, which has been reported to be a consequence of cyclin dependent kinase mediated phosphorylation of E1 [16, 17]. Mutations in the viral E1 or E2 genes result in integration or loss of the viral genome [18].

Eukaryotic origin licensing encompasses a highly orchestrated series of precise steps that limits firing to once per cell cycle [19]. DNA synthesis begins when the seven-subunit origin recognition complex (ORC) assembles on DNA. In contrast to the ~30 base pair (bp) PV origin, the sequence requirements for metazoan replication origins definition are poorly defined, and these may span chromosomal regions of perhaps 100,000 kb [20–23]. Mammalian replication initiates at the predicted 10^4^–10^5^ origins in a processive cascade that is coordinated by loading of the pre-replication complex (pre-RC) onto chromatin [24–26]. The experiments performed herein include chromosomal regions that are recognized to contain potential origins for comparisons to their PV counterparts operative in the same cell. The ORC1 protein is the only ORC member to contain a bromo-adjacent homology domain (BAH) necessary for interaction with DNA replication origins [27]. ORC1 is bound to DNA during G1 and released during S-phase and DNA synthesis, while ORC proteins 2–6 may be attached to DNA throughout the majority of cell cycle. ORC2 association with chromatin is regulated by cell cycle dependent phosphorylations at threonines 116 and 226, which lead to its dissociation from chromatin during the transition from S to M while still in complex with the other ORC proteins [28]. After ORC binding to chromatin in late M through G1, the multiple mini-chromosome maintenance complex (Mcm2-7) DNA helicase is loaded onto chromatin following binding of Cdc6 to ORC.

Very little is known of which host replication factors are required during each stage of the viral replicative program. We describe here that BPV and HPV E2 proteins bind to ORC2, however, this pre-RC factor is not present at the viral replicon and its depletion did not suppress viral replication but instead increased the presence of E1 and E2 at the viral origin. This led to the hypothesis that E2 association with ORC2 regulates viral and cellular origin loading such that at low levels of E2, its complex with ORC2 suppresses viral replication. When high levels of E2 are achieved in the differentiation dependent viral genome amplification stage, E2 restricts cellular origin recognition by ORC, which would otherwise complete for factors necessary for viral DNA synthesis. These results illustrate a novel mechanism by which PV E2 orchestrates the viral genome replication program in a stratifying epithelial environment.

## Results

### ORC2 protein in complex with BPV-1 and HPV-31 E2 proteins

**Fig 1:**
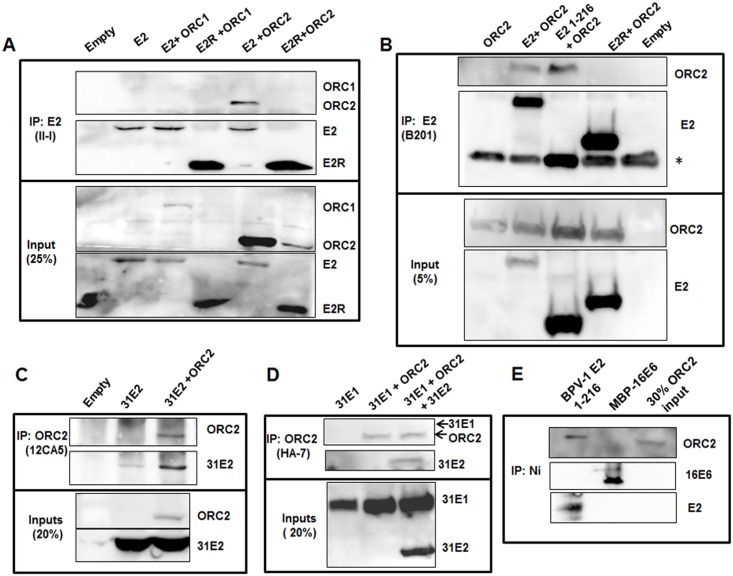
ORC2 co-immunoprecipiates with PV E2. (A) FLAG-mouse ORC2 or ORC1 were transfected into C33A cells with either BPV-1 E2 or E2R (aa 162–410). 24 hr later, E2 immunoprecipitations (II-1 antibody) were blotted with E2 B201 or M2 antibodies. (B) ORC2 interacts with the BPV-1 E2 transactivation domain (TAD, aa 1–216). FLAG-mouse ORC2 was transfected into C33A cells with BPV-1 E2, TAD or E2R. 24 hr later, groups without full length E2 were treated with 10 μM MG132 for 6 hr to maintain input protein levels of ORC2. B201 antibody immune complexes were blotted with B201 and M2 antibodies. The TAD domain of E2 is the same size as light chain IgG as indicated by * in the Fig 1B. (C) HA-ORC2 was transfected into 293TT cells along with FLAG-HPV-31 E2. HA antibody immunoprecipitates were blotted with HA-7 and M2 antibodies. (D) HA-ORC2 was transfected into 293TT cells along with FLAG-HPV-31 E1 and FLAG-HPV-31 E2. HA antibody immunoprecipitations were blotted with ORC2 and FLAG antibodies. ORC2 was not detected in the inputs but was detected in the pull down. HA-ORC2 protein is approximately 70 kDa and FLAG-31E1 protein is approximately 75 kDa. (E) Human ORC2 protein was incubated with 6-His-BPV E2:1–216 and MBP-6-His-HPV-16 E6. Nickel complexes were blotted with E2 (B201), E6, and ORC2 antibodies.

**Fig 2:**
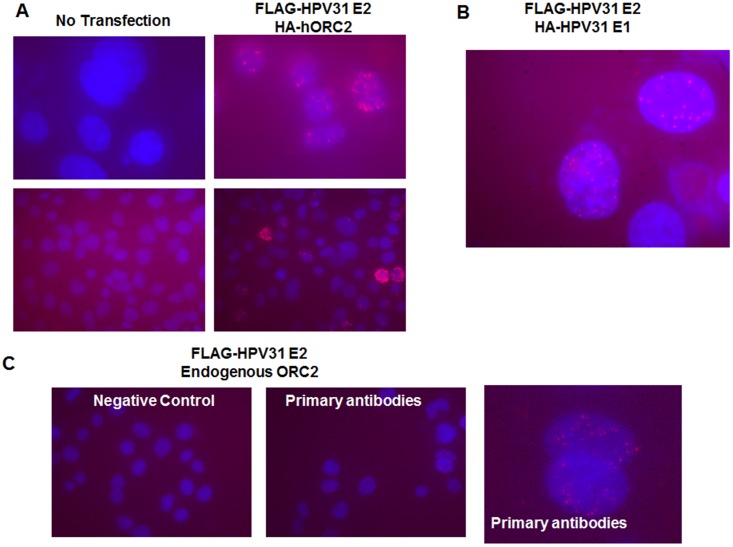
ORC2 co-localizes with HPV-31 E2 *in vivo*. (A) *In-situ* proximity ligation assay (PLA) between FLAG-HPV-31 E2 and HA-hORC2. Fluorescent spots indicate interaction based DNA amplification occurring in the nucleus (stained blue). The no transfection group was used as a negative control with these antibodies. (B) PLA between FLAG-HPV-31 E2 and HA-HPV-31 E1. (C) PLA between FLAG-HPV-31 E2 and endogenous ORC2. Secondary antibodies only were used as a negative control.

The current study began as we questioned whether ORC proteins assemble at the PV origin. While E1 and E2 proteins are sufficient to activate the PV ori, it is possible that a pre-ORC factor also participates in viral ori licensing. For example, the Epstein-Barr Virus (EBV) EBNA-1 protein binds to and recruits ORC2 to oriP [29]. This leads to entry of ORC2-4 along with the helicase component MCM2 [30]. E2 has structural and functional similarities to EBNA-1 [31, 32], however EBV does not utilize a viral DNA helicase that resembles E1 at oriP. We initiated this survey using immunoprecipitation of the BPV-1 E2 protein followed by immunoblotting for the pre-RC mammalian factors ORC1 and ORC2. C33A cells were transfected with plasmids expressing BPV-1 E2 or the truncated E2R that lacks the 160 initial amino acids of the TAD [33] along with mouse FLAG-ORC1 or FLAG-ORC2. Full length BPV-1 E2 but not E2R co-immunoprecipitated ORC2 protein but not ORC1 (Fig 1A). Next we determined whether the TAD was sufficient for association with ORC2. BPV-1 E2 full length, TAD, and E2R along with mouse ORC2 were expressed in C33A cells. Since full length BPV-1 E2 increases transcription of most co-transfected genes [34], cells were treated with 10 μM MG132 for 6 hr except for those expressing full length E2 to achieve comparable ORC2 protein levels among all the experimental groups. Both E2 and its TAD individually co-immunoprecipitated ORC2, whereas the E2R form did not (Fig 1B). In analogous experiments, HA-tagged human ORC2 (HA-hORC2) co-immunoprecipitated with FLAG-HPV-31 E2 (Fig 1C) but not with the HPV-31 E1 protein (Fig 1D). The presence of HPV-31 E1 did not interfere with ORC2 pull down with HPV-31 E2 (Fig 1D). To diminish the possibility that other mammalian factors bridge E2 to ORC2, baculovirus lysates containing human ORC2 protein were combined with bacterially expressed 6-histidine tagged BPV-1 E2 TAD or control HPV-16 E6 proteins. Nickel bead collection of these his-tagged proteins showed that ORC2 specifically interacted with the E2 TAD (Fig 1E), implying that in the absence of other mammalian replication factors, ORC2 may bind directly to E2. Subsequently we confirmed the protein-protein interaction between E2 and ORC2 *in vivo* using *in-situ* proximity ligation assays (PLA). As a positive control, we observed fluorescent puncta in cells transfected with FLAG-HPV-31 E2 and HA-HPV-31 E1 (Fig 2B). C33A cells transfected with FLAG-HPV-31 E2 and HA-hORC2 showed fluorescent spots compared to untransfected cells (Fig 2A), indicating association between two proteins *in vivo*. We also detected PLA interaction between FLAG-HPV-31 E2 and endogenous levels of ORC2 (Fig 2C).

### ORC2 and PV genome replication

**Fig 3:**
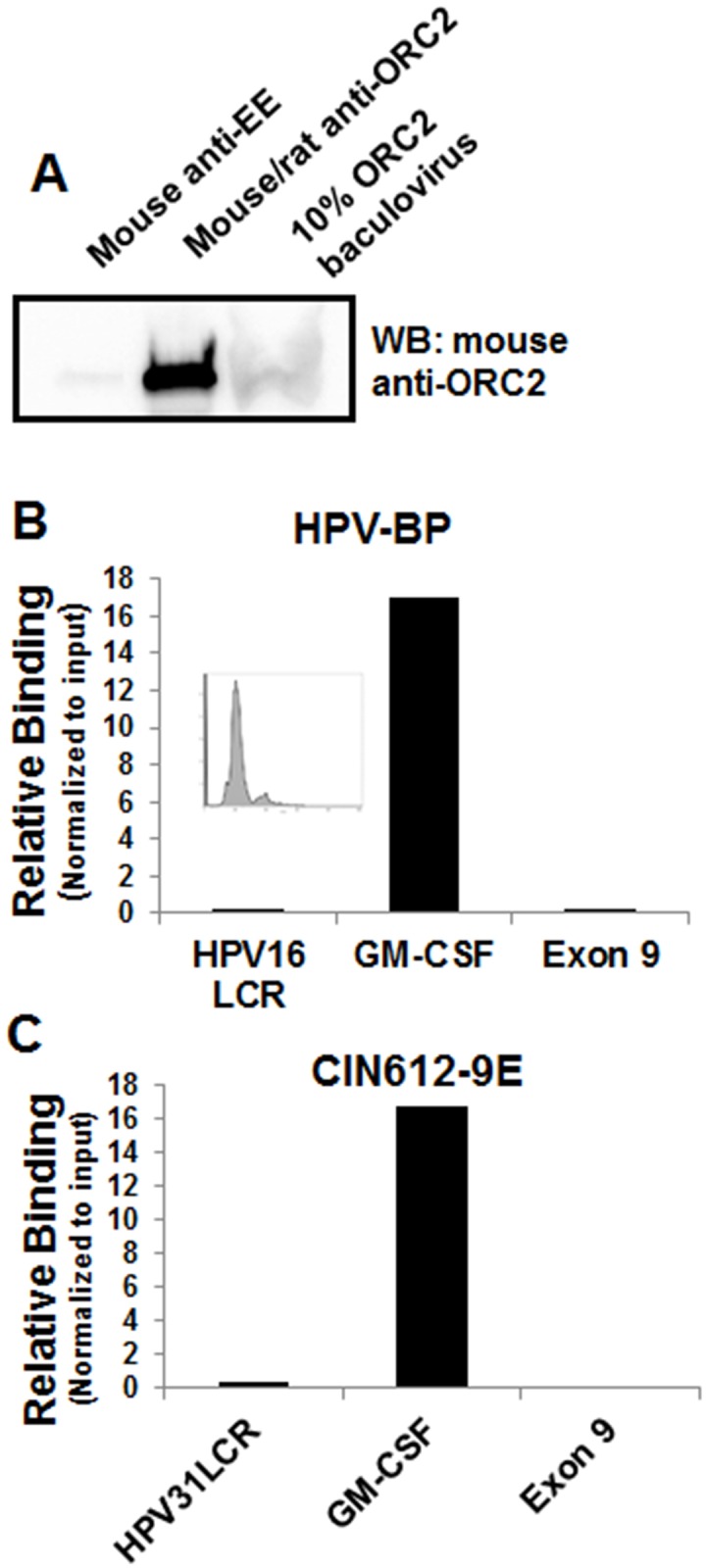
ORC2 is present on a known mammalian origin but not the HPV ori. (A) Baculovirus expressing human ORC2 protein was added to 0.5% NP-40 lysis buffer containing protein A and protein G agarose bead slurry, and either mouse anti-EE or an equal mix of mouse anti-ORC2 (MBL) and rat anti-ORC2 (Cell Signaling) antibodies. IPs were loaded on a SDS page gel and ORC2 was detected with the mouse anti-ORC2 antibody (MBL) by immunoblotting. (B) HPV-BP and (C) CIN612-9E cells were synchronized with 2.5 mM double thymidine. Inset diagram shows flow cytometry based cell cycle analysis. ChIP was performed using mouse anti-ORC2 antibodies (MBL). Real-time PCR was completed with primers to the HPV-16 or HPV-31 LCR, a known mammalian origin of replication (GM-CSF), and Exon 9, a non-specific DNA region used as a negative binding control. EE (non-specific IgG) did not bind to these regions of DNA in these experiments and is not shown here.

**Fig 4:**
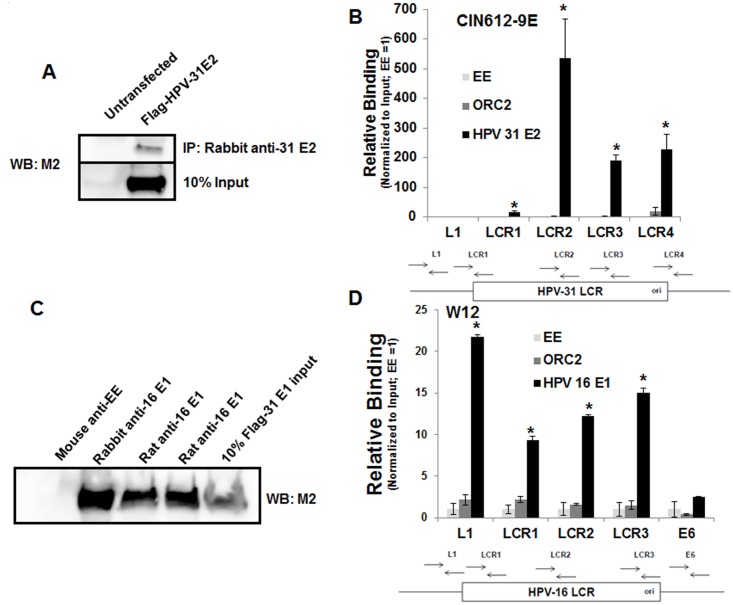
ORC2 is not present at the HPV-31 or HPV-16 viral origin. (A) C33A were transfected with the FLAG-HPV-31 E2 expression vector. 24 hours later cells were lysed (0.5% NP-40). The lysate from cells untransfected and cells transfected with FLAG-HPV-31 E2 were added to lysis buffer containing protein A and protein G bead slurry and rabbit anti-HPV-31 E2 serum. IPs were immunoblotted with M2 antibodies. (B) CIN612-9E cells were synchronized with 2.5 mM double thymidine. ChIP experiments were performed using mouse anti-EE, an equal mix of rat and mouse anti-ORC2 (Cell Signaling/MBL) or rabbit anti-HPV-31 E2 antibodies. Real-time PCR was performed with primers listed in Materials and Methods located in and around the HPV-31 LCR. The locations of the primers are shown below the horizontal axis with the ori labeled. E2 binding sites are located between the LCR2 and LCR4 primer sets. Ct values are normalized to input and EE was set to equal 1. Values are expressed as mean +/- SEM. * p-value ≤ 0.05 compared to EE. (C) C33A cells were transfected with FLAG-HPV-31 E1 expression vector and lysates were immunoprecipiated with mouse anti-EE, rabbit anti-16 E1, and rat anti-16 E1 antibodies and then immunoblotted with M2 antibodies. (D) W12 cells were synchronized with 2.5 mM double thymidine. ChIP experiments were performed using mouse anti-EE, equal mix of rat and mouse anti-ORC2 antibodies (Cell Signaling/MBL), or rat anti-HPV-16 E1 antibodies. Real-time PCR was performed with primers listed in Materials and Methods located in and around the HPV-16 LCR. The locations of the primers are shown below the horizontal axis with the ori labeled. E2 binding sites are located between the LCR3 and E6 primer sets. Ct values are normalized to input and EE was set to equal 1. Values are expressed as mean +/- SEM. * p-value ≤ 0.05 compared to EE.

Because E2 engages the viral ori, the expectation was that ORC2 would be present near the PV ori or the flanking sequences of the long control region (LCR). After confirming that commercial ORC2 antibodies immunoprecipitate the native ORC2 protein using baculovirus expressed human ORC2 lysate (Fig 3A), we performed a series of chromatin immunoprecipitation (ChIP) experiments using HPV-BP and CIN612-9E cells, which maintain low copy HPV-16 and HPV-31 episomes respectively. These cell lines, expressing E1 and E2 from native viral promoters, were synchronized to G1/S with double thymidine to capture putative ORC complexes at or near the PV ori. Repeated ChIP assays failed to detect endogenous ORC2 near the HPV-16 or HPV-31 LCR, while ORC2 was detectable near the mammalian origin of replication present within the GM-CSF gene (Fig 3B and 3C). A 6 kb region upstream of the MCM4 upper regulatory region (Exon 9) and the associated primers, previously reported to be free of ORC2 [35], served as a negative control. Commercially available ORC2 antibodies do not have high affinity for mouse ORC2, prohibiting analogous ChIP experiments in C127 cell lines that stably replicate BPV-1 episomes. Because ORC2 was not present near the viral ori, other regions within and flanking the LCR were probed for the presence of ORC2 and compared to antibodies directed against the viral E1 or E2 proteins as positive controls. ChIP was performed for HPV-31 E2 and ORC2 using lysates from CIN612-9E cells enriched in G1/S with double thymidine. In these experiments, we used an anti-peptide antibody to HPV-31 E2 (epitope aa 97–109; Fig 4A) and HPV-16 E1 antibodies confirmed to cross-react with the highly conserved HPV-31 E1 protein (Fig 4C). Target DNA immunoprecipitation with HPV-31 E2 was at least 15 fold greater than with negative control EE antibodies, while ORC2 could not be detected at these regions (Fig 4B). HPV-31 E2 enrichment was greatest near the region of the E2 binding sites between the LCR2 and LCR4 primer sets. To perform analogous experiments with a second HPV genotype, we used W12 cells that contain HPV-16 episomes. Consistent with our previous results, these ChIP reactions demonstrated the presence of HPV-16 E1 at the LCR while ORC2 was not detected at this location in the HPV genome (Fig 4D).

Previous studies observed that E1 and E2 co-expression leads to formation of nuclear foci in CV-1, C33A, and primary human foreskin keratinocyte (HFK) cells in the presence and/or absence of the HPV origin [8, 36–38]. These are considered to be ‘replication centers’ where E2 and E1 assemble on viral genomes. Several DNA repair proteins have been shown to accumulate at these foci, including ɣH2AX, pATM, pChk2, NBS1, and Rad51 [39, 40]. While the ChIP data implied ORC2 does not directly participate in viral ori licensing, we questioned whether it was present at these nuclear foci. CV-1 cells were transfected with expression vectors for HPV-31 E1, HPV-31 E2, and YFP-ORC2 with or without an HPV-31 ori containing plasmid. HPV-31 E2 and YFP-ORC2 expression alone are both diffusely nuclear. YFP-ORC2 did not co-localize with E1 and E2 nuclear foci in the presence (S1A–S1C Fig) or absence of the HPV-31 origin (S1D–S1F Fig). We also confirmed these observations using BPV E1 and E2 proteins, which were detected in discrete nuclear spots, while ORC2 was dispersed throughout the nucleus (S2 Fig). This observation is consistent with our ORC2 co-IP data in which ORC2 interacted with E2 but E1 was not in this complex (Fig 1D).

**Fig 5:**
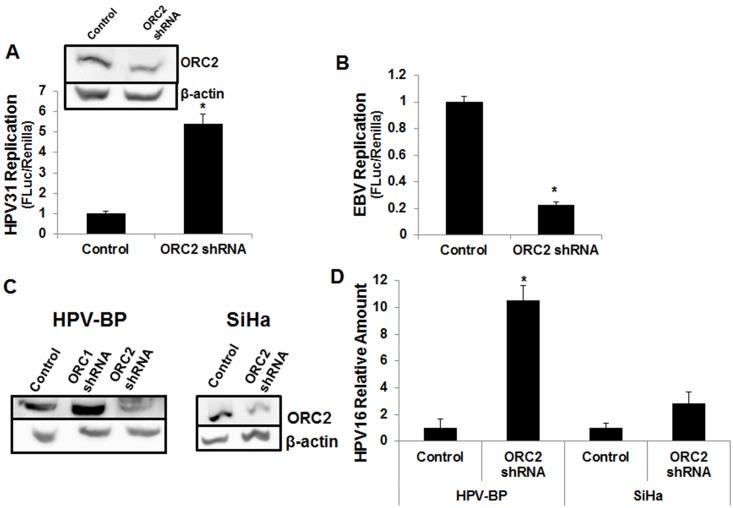
ORC2 knockdown enhances PV replication. (A) Replication luciferase assays were completed with pFLORI31 and ORC2 depletion. Values are expressed as mean +/- SEM. * p-value ≤ 0.05. ORC2 shRNA decreased ORC2 protein expression in C33A cells. (B) ORC2 shRNA decreased EBNA-1 based replication using the pREP4 plasmid (EBNA-1 based replication) in C33A cells. (C) ORC2 shRNA decreased ORC2 protein expression in HPV-BP and SiHa cells. (D) ORC2 knockdown increased HPV replication in cells containing HPV-16 episomes (HPV-BP) but not in cells with integrated HPV-16 (SiHa). HPV-BP and SiHa cells were transfected with 5.5 μg of shRNA hairpins. 7d later, cells were harvested and HPV-16 copy number (HPV-16 LCR) as normalized to GM-CSF ori #2. This experiment was performed three times with a representative image presented here.

Because ORC2 co-immunoprecipitated with E2 yet was not detectable at the viral ori or at viral replication foci, this inferred that ORC2 is not necessary for viral replicon licensing. To provide experimental evidence for this, we performed transient replication assays in which firefly luciferase activity is proportional to episomal copy number as corroborated by PCR for viral DNA content [41–45]. Further confirmation of this assay is sensitivity to DNA replication inhibitors [46]. For the analyses, we normalized firefly luciferase levels to co-transfected renilla levels to control for non-specific transcriptional effects. Due to relatively high levels of E1 and E2 protein expression, this experimental transient transfection model likely reflects the amplification stage of viral genome replication [47]. ORC2 depletion resulted in increased replication of the luciferase-linked HPV-31 ori plasmid driven by co-transfected E1 and E2 (Fig 5A). ORC2 knockdown did not change luciferase levels when the ori was transfected with E1 or E2 alone (S3A Fig). Using BPV-1 E1 and E2 with a luciferase-reporter ori plasmid, ORC2 knockdown increased BPV-1 replication though not to same extent as with HPV-31 (S3B Fig). The non-specific transcriptional activity of BPV-1 E2 may contribute to its higher basal luciferase levels [48]. The ORC2 shRNA hairpin had no effect on luciferase activity with co-expression of replication incompetent E2R with BPV-1 E1.

We also investigated the consequences of ORC2 depletion on the HPV replication at endogenous E1 and E2 protein levels maintained in monolayer cultures by co-transfecting the ORC2 shRNA together with the HPV-31 ori reporter plasmid into CIN612 cell lines. These conditions represent the maintenance phase of the viral replication program. The reporter plasmid system was necessitated because direct measurement of viral copy number by quantitative PCR would include the large number of CIN612-9E cells that were not transfected with the shRNA. Consistent with our previous studies, ORC2 shRNA induced increased luciferase production and displayed a corresponding reduction of ORC2 protein (S3C Fig). for comparison, we investigated the consequences of ORC2 depletion on the pREP4-EBNA1, an EBV based episomal oriP plasmid containing the EF1α promoter and luciferase reporter gene. Transfection of the ORC2 shRNA suppressed EBNA1 induced replication (Fig 5B), consistent with the observation that EBV replication is ORC2 dependent [29]. As ORC2 shRNA knockdown had the opposite effect on luciferase levels with the EBV reporter compared to the HPV reporter, we concluded that the increased luciferase levels in the HPV system was not due to a direct effect of ORC2 shRNA on luciferase protein expression but reflective of PV copy number. In more efficiently transfected HPV-BP cells, knockdown of ORC2 showed a ten fold increase in HPV-16 DNA episome number measured by quantitative PCR with primers specific to the HPV-16 LCR (Fig 5C and 5D). In contrast, ORC2 knockdown did not produce a significant change in HPV-16 DNA content in SiHa cells, which contain a single integrated copy of the HPV-16 genome. While it might be argued that ORC2 knockdown might change luciferase mRNA expression or stability, these experiments demonstrated the same effects on cells with endogenous HPV genomes.

**Fig 6:**
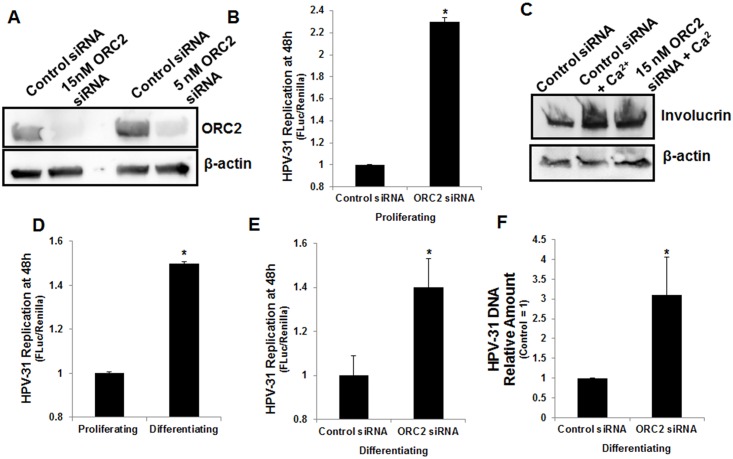
ORC2 silencing enhanced PV replication in proliferating and differentiating CIN612-9E cells. (A) ORC2 siRNA duplexes decreased ORC2 protein levels at a concentration of 15 or 5 nM after 48 h. (B) CIN612-9E cells were transfected with RLuc and pFLORI31. 48h later cells were lysed and luciferase activity measured. Values are expressed as mean +/- SEM. * p-value ≤ 0.05. (C) CIN612-9E cells were transfected with 15 nM ORC2 and differentiated in 10% FBS DMEM + 2 mM CaCl_2_ for 48h. Involucrin and β-actin levels were analyzed. (D) CIN612-9E cells were transfected with RLuc and pFLORI31. Five hours later, the transfection was removed and cells were placed in either E-medium or 10% FBS DMEM + 2 mM CaCl_2_. 48h later cells were lysed and luciferase activity measured. Values are expressed as mean +/- SEM. * p-value ≤ 0.05. (E) CIN612-9E cells were transfected with 15 nM control or ORC2 siRNAs, RLuc and pFLORI31. Five hours later, the transfection was removed and cells were placed in 10% FBS DMEM + 2 mM CaCl_2_. 48h later cells were lysed and luciferase activity measured. Values are expressed as mean +/- SEM. * p-value ≤ 0.05. (F) CIN612-9E cells were transfected with 15 nM control or ORC2 siRNAs and placed in 10% FBS DMEM + 2 mM CaCl_2_ for 72 h. DNA was isolated and HPV-31 DNA content was measured by RT-PCR and normalized to β-actin levels. The ORC2 siRNA group was normalized to the control siRNA group (control siRNA = 1). Values are expressed mean +/-. * p-value ≤ 0.05.

**Fig 7:**
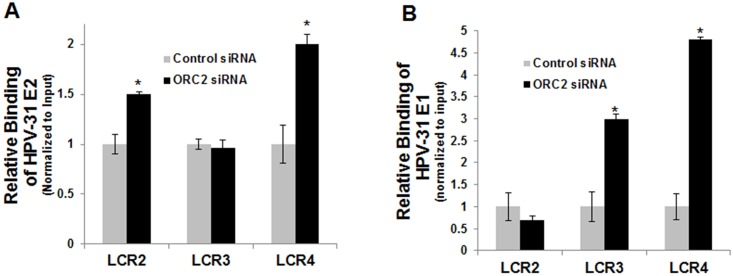
ORC2 depletion increases E1 and E2 occupancy at the viral origin in CIN612-9E cells. CIN612-9E cells were transfected with 30 nM of control or ORC2 siRNAs for 48 h. ChIP experiments were performed using (A) rabbit anti-HPV-31 E2 antibodies or (B) rat E1 antibodies. Real-time PCR was performed with primers listed in Materials and Methods located in HPV-31 LCR previously found to be enriched for E2 binding. E2 binding sites are located between the LCR2 and LCR4 primer sets. Ct values are normalized to input and values are expressed as mean +/- SEM. * p-value ≤ 0.05 compared to control siRNA.

The decrement in ORC2 protein levels following ORC2 shRNA was relatively small, as we assumed that a dramatic reduction in ORC2 levels would be detrimental to cell proliferation. Nonetheless, an ORC2 siRNA duplex was obtained to attempt more effective reduce the ORC2 expression in CIN612-9E cells. The ORC2 siRNA caused a more dramatic reduction of ORC2 levels compared to the previously used ORC2 shRNA at 48 h (Fig 6A). ORC2 silencing had no effect on the cell cycle profile at 48 h (S4A Fig). We then tested the consequences of ORC2 depletion on HPV-31 replication in a model of replication maintenance. First, CIN612 cells were transfected with the HPV-31 ori reporter plasmid in the absence or presence of ORC2 siRNA. As seen in Fig 6B, ORC2 depletion enhanced luciferase levels at 48h. In the following series of experiments, we evaluated the effects of ORC2 depletion on viral replication during keratinocyte differentiation [49, 50]. CIN612 cells expressing endogenous levels of HPV-31 E1 and E2 were transfected with the HPV-31 ori reporter plasmid. To induce differentiation, cells were placed in 10% FBS DMEM + 2 mM CaCl_2_ for 48 h. Levels of the differentiation marker involucrin protein were elevated after calcium treatment (Fig 6C). As shown in Fig 6D, luciferase expression increased approximately 1.5 fold during differentiation, which is consistent with previous studies using Southern blotting to measure copy number at 48 h in CIN612-9E cells [40, 51]. Subsequently, control and ORC2 siRNAs were transfected into CIN612 cells along with the HPV-31 ori reporter plasmid in the presence or absence of 48 h calcium and normalized to renilla levels. Consistent with the shRNA experiments, ORC2 silencing enhanced HPV-31 ori luciferase expression (Fig 6E). Finally, we measured HPV-31 DNA genome levels in CIN612 cells after ORC2 depletion and calcium induced differentiation. HPV-31 DNA content was significantly higher with ORC2 knockdown compared to the control at 72 h (Fig 6F). Consistently, the ORC2 depleted cells showed increased HPV-31 E2 and E1 occupancy near two of the three viral origin regions tested by ChIP assay (Fig 7).

### E2 over-expression decreases ORC2 loading onto known mammalian origin

**Fig 8:**
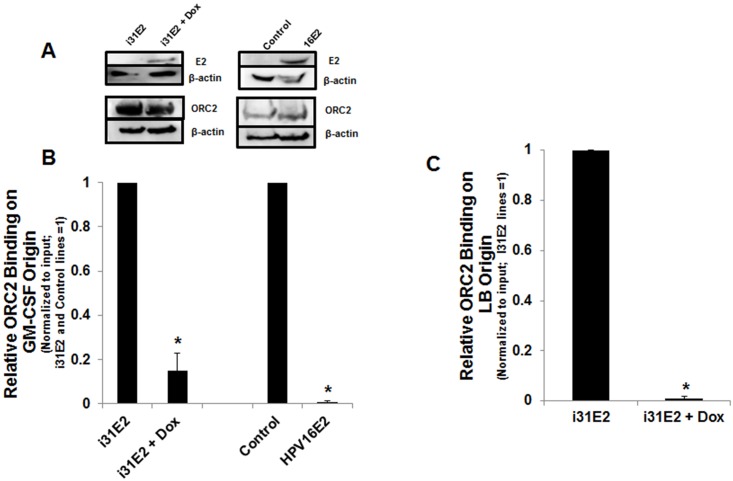
Overexpression of E2 decreases ORC2 occupancy at a known mammalian origin. (A) Lysates (1% NP-40) for i31E2 cells ± Dox and control/HPV-16E2 U2OS cell lines were blotted with FLAG-M2 (HPV-31 E2), TGV261 (HPV-16 E2), ORC2 (MBL) and β-actin antibodies. (B,C) TRE-x U2OS cells containing pcDNA4/TO-FLAG 31E2 (i31E2, ± 48h Dox treatment) and Control and 16E2 U2OS cells were treated overnight with 2.5 mM thymidine, released the next day for 6h, followed by 2.5 mM thymidine treatment overnight. ChIP was performed with an equal mix of mouse/rat-ORC2 antibodies (MBL/Cell Signaling) on the GM-CSF ori (B) or the lamin B2 (LB) ori (C). ChIP experiments were performed at least three times. The Ct values for ORC2 binding were first normalized to input for each cell line. ORC2 levels for the E2 expressing cell lines were normalized to the control cell lines (no E2). Values are expressed as mean +/- SEM. * p-value ≤ 0.05 compared to control cell line.

Taken together, these results implied that ORC2 is not necessary for HPV ori licensing, so the logical question became what is the biological significance of E2 binding to ORC2. A clue is that high level expression of the HPV E2 protein has been reported to inhibit cellular proliferation [52, 53]. This led us to hypothesize that E2 association might interfere with ORC2 function. To test this, we queried the effects of E2 expression on ORC2 occupancy at a frequently utilized mammalian origin. These ChIP experiments are challenging since it is well established that origin utilization is variable in both site usage and timing [54, 55] and because of the magnitude and repetitive composition of the human genome [56]. These experiments included U2OS T-REx cells with a doxycycline-inducible HPV-31 E2 expression cassette (i31E2) and U2OS cells that stably express the HPV-16 E2 protein (16E2). ORC2 protein levels were unaltered in the presence of E2 (Fig 8A). It was reported that HPV-16 E2 expressing U2OS cells are capable of progression into and through S-phase although this occurs at a slower rate [57]. We analyzed the cell cycle between the E2 expressing cell lines and their controls and found no difference between their cell cycle profiles (S4B Fig). U2OS, 16E2, and i31E2 cells were enriched in G1/S phase content and ChIP assays were performed with ORC2 and control antibodies. Occupation of ORC2 at the GM-CSF origin was reduced in cells expressing HPV-31 E2 and HPV-16 E2 (Fig 8B). The presence of ORC2 on the lamin B2 origin [35] was also decreased in i31E2 cells following dox-induced HPV-31 E2 expression (Fig 8C).

## Discussion

For the timely and efficient duplication of an entire metazoan genome, DNA synthesis must begin at an estimated 10–100,000 replication origins that must be activated once and only once per cell cycle [20]. Ori firing is coordinated by ORC binding followed by a cascade in which the MCM2-7 hexamer, Cdc6, and Cdt1 assemble during G1 and become activated by protein kinases during S-phase [58]. While metazoan origins span tens of thousands of kilobases, small viral genomes cannot accommodate such vast sequences. Furthermore, papillomaviruses do not encode any DNA polymerases and yet persistently infect and autonomously replicate their genomes in host cells. Functionally, the PV origin consists of recognition sites for the high affinity and high specificity E2 protein, a contiguous binding site for E1, and a flanking A-rich tract. Upon entry of a single papillomavirus genome into a basal epithelial cell nucleus, it undergoes replication to a few copies. The viral E1 and E2 proteins are not present in the virion, so viral transcription with accompanying synthesis of these proteins is assumed to occur very early after infection. A recent study found that replication begins at the viral ori and proceeds through a theta structure model, although subsequent replication appeared to be unidirectional and did not initiate at a specific site [59]. Viral copy number is maintained in proliferating basal keratinocytes and *in vitro* cultured cells that carry PV episomes, which presumably requires viral origin licensing during G1/S after which the replicated genomes partition without triggering the mitotic spindle dependent checkpoint [60]. Viral genome segregation involves E2 interaction with Brd4 and ChlR1 [6, 61, 62]. The genome amplification stage occurs in response to an unknown differentiation associated cue. Activation of the ATM/ATR DNA damage pathway has been shown to stimulate viral genome amplification [2, 36] but is not necessary for episome maintenance [63]. TopBP1, a protein involved in the DNA damage response pathway, is essential for initiation of viral replication and may play a crucial role for genome amplification [64, 65]. Our goal is to characterize the cellular replication factors that are necessary for each stage of the PV tri-phasic replicative program.

In experiments to investigate involvement of ORC factors in PV replication, we discovered that ORC2 co-immunoprecipitates with BPV-1 and HPV-31 E2. The E2 protein regulates viral transcription by interacting with cellular factors and targeting these to the viral genome via its C-terminal DNA binding domain [8]. Its N-terminal trans-activation domain (TAD) was sufficient for ORC2 association. We were able to detect by ChIP endogenous levels of HPV-31 E2 and HPV-16 and -31 E1 proteins at the viral ori using a cell line that stabily maintains HPV episomes. Unexpectedly, ORC2 was not present at the HPV origin region in these cells under conditions where ORC2 was detectable at a commonly utilized cellular origin. It seems unlikely these antibodies might not access the ORC2 complex at the viral DNA. These data inferred that ORC2 is not necessary for viral origin licensing. Consistent with this observation, YFP-tagged ORC2 protein was not visualized at E1 and E2 nuclear replication foci but instead appeared diffusely throughout the nucleus. However, we cannot exclude the possibility that the ORC2 epitope is not available or that a few molecules of ORC2 protein are present in these foci.

An RNAi ORC2 knockdown strategy was pursued with the goal of reducing but not eliminating this key pre-RC factor. ORC2 depletion increased viral copy number in transient replication assays and in keratinocytes that maintain episomal HPV-16 genomes relative to genomic DNA content. ORC2 depletion had no effect on HPV content in SiHa cells with an integrated HPV-16 genome. Consistently, ORC2 knockdown increased HPV-31 DNA content in HPV-31 episomal cell lines. In experiments in which ORC2 expression vectors were co-transfected along with a PV-replicon reporter or E2 vector, the cells were unable to be established, indicating that over-expression of ORC2 is deleterious for cell proliferation, and hence effects on viral replication could not be studied. We found that short term ORC2 knockdown did not alter the cell cycle prolife, implying sufficient levels of ORC2 are present to sustain growth. Previous reports have found depletion of ORC2 has different outcomes on the cell cycle dependent on cell type. For example, ORC2 siRNA decreased S-phase DNA content in MCF10A breast cancer cells [66]. In HeLa cells, ORC2 depletion showed a slower S-phase and an increased M-phase [67]. However in HCT116 with a hypomorphic ORC2 mutation that expressed 10% of ORC2 compared to wild-type replicated normally once is S-phase [68]. There is evidence for post-translation modifications of ORC2, which may regulate ori licensing [28].

Utilization of re-replication (pre-RC) proteins has been discovered with several large DNA viruses including two gamma herpesviruses. EBV genomes utilize the mammalian replication licensing mechanism for conservation of viral copy number in latently infected cells. EBNA1 binds to and recruits ORC2 to the EBV oriP [29, 30]. Consistent with its role in EBNA1 regulated EBV replication, our ORC2 knockdown experiments disclosed a comparative decrease in EBNA-induced transient replication. ORC2 was reported to bind to Kaposi’s sarcoma-associated herpesvirus (KSHV, HHV-8) terminal repeats where LANA dependent replication initiates [69]. Depletion of ORC2 was reported to decrease KSHV latent replication [69]. In contrast and similar to our observations, reduced levels of ORC2 increased lytic replication of beta herpesvirus human cytomegalovirus (HCMV, HHV-5) [70], which conceptually resembles PV amplification that occurs in differentiated epithelial cells.

We conclude that PV ori licensing does not require ORC2. The data shown here indicate that ORC2 suppresses PV genome replication during its maintenance phase. Levels of the E2 protein are very low in the maintenance phase of the viral life cycle such that the ORC2 protein may interfere with E2 loading at the viral origin. Increased viral replication following ORC2 depletion is likely due to the increased occupancy of E1 and E2 detected at the viral origin.

The ratio of ORC2 to E2 changes during the viral amplification stage, which resemble a G2/M arrest state imposed by high levels of the HPV E4 protein in the upper epithelium [71, 72]. While this has been proposed to allow for continued viral DNA synthesis in the absence of cell cycle progression, tens of thousands of human origins persist and might be licensed and activated. Our hypothesis is that the high levels of E2 that occur in differentiated epithelial strata [49, 50] inhibit ORC2 function and thereby restrict firing of host replicative origins that would otherwise effectively compete for replication enzymes necessary for viral genome amplification. Several studies have shown that over-expression of E2 induces growth arrest in both HPV positive and negative cell lines [47, 53, 73–75]. The E2-mediated growth arrest in HPV positive cells is mediated through expression of E6 and E7 [76], but the mechanism in HPV negative cells is unclear. In HaCat cells, over-expression of HPV-16 E2 induced apoptotic cell death, however, surviving cells maintained long-term low expression of E2 and appeared to be terminally differentiated [77]. Primary human foreskin keratinocytes infected with HPV-31 E2 adenovirus arrested in S-phase [52]. Inducible expression of HPV-31 E2 protein in U2OS cells resulted in reduced ORC2 bound at the GM-CSF ori. The cell cycle profile in the cell lines with E2 over-expression did not change suggesting that reduced ORC2 bound at the origin was not due to cell death.

These experiments illustrate the dual nature of the E2-ORC2 interaction and how the virus senses and manipulates its cellular environment. At low levels of E2 present in basal cells, ORC2 inhibits viral replication and thereby prevents a lytic infection. During epithelial differentiation when elevated levels of E2 are achieved, its complex with ORC2 restricts host cell origin licensing thus promoting replication of the HPV amplicon.

## Materials and Methods

### Plasmids and antibodies

Codon optimized FLAG HPV-31 E2 [36] was cloned between the BamHI and HindIII sites of pcDNA3. Codon optimized triple FLAG HPV-31 E1 [41], pCG-BPV-1 E1 Eag123 [78], the ori-luciferase plasmids for the PV transient replication assay [41], pLKO.1 ORC2 shRNA [64], YFP-ORC2 [40] used were previously reported. For shRNA experiments, pCMV-GIN-Zeo (Open Biosystems) was used as the control plasmid. Human ORC2 cDNA was cloned between the NheI and ApaI sites in pcDNA3 containing a HA tag. Mouse ORC1 and ORC2 cDNA were amplified by PCR from mouse cDNA with FLAG tags and cloned into the MluI and SalI sites of the pCI backbone. The BPV-1 expression vectors and GST vectors have been described elsewhere [10, 79]. pREP4 which contains the EBV oriP and EBNA1 was modified to contain a E1F-α minimal promoter and used to measure EBV replication. To generate inducible HPV-31 expression, codon optimized FLAG HPV-31 E2 was cloned into the HindIII and ApaI sites of pcDNA4/TO-luc creating pcDNA4/TO-FLAG 31 E2.

The following antibodies were used: Mouse anti-FLAG (M2; Sigma), mouse anti-HA (HA-7; Sigma 3F10; Roche, 12CA5), mouse anti-human ORC2 (MBL), rat anti-human ORC2 (Cell Signaling), mouse anti-HPV-16 E6 (Arbor Vita), mouse anti-EE and anti-β-actin (Sigma). BPV-1 E2 was identified with B201 (a mouse monoclonal antibody with an epitope between amino acids 160–220) and II-1 rabbit antisera [12]. The sheep anti-HPV-16 E2 antibody was described elsewhere [62]. With the prior approval of the Johns Hopkins University Animal Care and Use committee (protocol RA14M47), two Lewis rats were immunized three times with 50 μg of GST-tagged E1 N terminal fusion protein (GST-E1N); (1–209 amino acids of HPV16 E1 protein) formulated with TiterMax (TiterMax USA, Inc.) adjuvant and the fourth immunization only GST-E1N was used. Three days following the final immunization, splenocytes were harvested, pooled and fused with SP2/0 myeloma cells using polyethylene glycol (PEG). Supernatants from wells containing viable hybridomas were screened at 10 to 14 days after fusion by ELISA using bacterially expressed GST-E1N and GST-tagged E2 (GST-E2) derived from HPV16 as a coating antigen. Selected positive clones that recognized GST-E1N but not GST-E2 were re-cloned three times by limiting dilution (1H4-4E-32.7.3.1 and 11B63G.6.1), and the antibodies secreted were purified using protein G Sepharose (GE Healthcare). An 18mer peptide array of E1 peptides within the 1–206 regions of HPV16, each overlapping by 15 residues was utilized to map the MAb epitopes by direct ELISA. Diluted hybridoma supernatants or purified antibodies were added to the blocked peptide-coated plates and incubated for 1 h at 37°C. Plates were washed three times with PBS containing Tween-20 again and then incubated with HRP conjugated goat anti-rat IgG for 60 min at 37°C. Unbound MAb was then washed away with 0.01% Tween 20 in PBS and signal detected with ABTS (Roche, Basel Switzerland). Anti-E1 MAb, 1H4-4E-32.7.3.1 isotype IgG2a, recognized only two overlapping E1 peptides comprising residues 4–21 and 7–24, its epitope likely within in 7–21 (TNGEEGTGCNGWFYV). Another anti-E1 Mab of isotype IgG2a, 11B63G.6.1, recognized 5 overlapping E1 peptides (4–21, 7–24, 10–27, 13–30 and 16–33) and, its epitope likely resides within residues 16–21 (NGWFYV). In addition, a rabbit was immunized 5 times with GST-E1N antigen (amino acid 1–209 of HPV16 E1) generated rabbit-anti-E1 serum (Generated by Proteintech Group, Inc.). Rabbit anti-HPV-31 E2 (10008) was generated to amino acids 97–109 (TSLELYLTAPTGC; manuscript in preparation).

### Cell culture

HEK293TT (from John Schiller and Chris Buck), CV-1 (from Douglas Lowy), SiHa (ATCC), U2OS T-REx (from Feng Yao), U2OS–HPV-16 E2 (from Iain Morgan), and C33A (from Douglas Lowy) were cultured at 37°C in Dulbecco’s Modified Eagle Medium (Life Technologies) with 10% fetal bovine serum (Atlas Biologicals) and penicillin/streptomycin (100 U/ml; Life Technologies) at 5% CO_2_. CIN612-9E (from Laimonis Laimins) a clonal cell line which maintains HPV-31 genomes were grown in E-medium with J23T3 fibroblast feeders (from Howard Green). W12 (from Margaret Stanley and Paul Lambert) a cervical carcinoma cell line with maintains HPV-16 genomes were grown in F-medium with J23T3 fibroblast feeders. HPV-BP cells (from Aloysius Klingelhutz) a clonal cervical keratinocyte cell line containing HPV-16 episomes were maintained in Keratinocyte SFM containing human recombinant Epidermal Growth Factor 1–53 (EGF 1–53), Bovine Pituitary Extract (BPE) and penicillin/streptomycin (100 U/ml; Life Technologies) at 5% CO_2_ [80]. High Five cells were grown in SF9 media (Life Technologies) at 30°C and 5% CO_2_. U2OS T-REx cells were transfected with 1 μg pcDNA4/TO-FLAG HPV-31 E2 using Lipofectamine 2000. 24 hrs later, 400 μg/ml of Zeocin was added for about two weeks until cell colonies were established. 800 ng/ml of doxycycline was added to the media to induce expression of FLAG-HPV-31 E2.

### *In-situ* proximity ligation assay (PLA)

*In-situ* PLA was performed using the PLA Red kit (Olink Biosciences). C33A cells were transfected with FLAG-HPV-31 E2 and HA-hORC2 or FLAG-HPV-31 E2 and HA-HPV-31E2 constructs. 24 h later, cells were fixed in 4% paraformaldehyde for 10 min, permeabilized for 15 min in 0.5% Triton-X 100/PBS, washed in PBS, blocked with 5% goat serum in 0.2% Triton-X 100/PBS, then incubated overnight with primary antibody combinations (E2–rabbit FLAG or ORC2–Mouse anti-human ORC2; or E2-M2 FLAG or E1-Rabbit 16E1) at 4°C. The PLA then followed the manufacturer׳s protocol. Briefly, cover slips were washed in buffer A, incubated with PLA probe PLUS and MINUS for one hour at 37°C, washed twice and the probes were ligated for 30 min. Amplification was performed for 100 min at 37°C, washed twice in buffer A and B. Cover slips were mounted in Duolink *in-situ* mounting media. Cells were analyzed with a Nikon microscope.

### Chromatin immunoprecipitation (ChIP)

Cells were synchronized (G1/S) using 2.5 mM thymidine for 16 hr, released for 6 hr, and retreated with 2.5 mM thymidine for 16 hr before harvest. For the ORC2 siRNA experiments, CIN612-9E were transfected with 30 nM of the siRNAs as described below. ChIP used the ChIP-IT express chromatin immunoprecipitation kit (Active Motif). Real time PCR was performed using Sso Fast Evagreen Mastermix (BioRad). Primers for the HPV-16 LCR in Fig 2 were 5’ GGGTGTGTGCAAACCGTTTTGGGTTA 3’ and 5’ CCGATTTCGGTTACGCCCTTAGTTT 3’ and primers for the HPV-31 LCR were 5’ CCTGCTCCTCCCAATAGTCAT 3’ and 5’ AAACGGACCGGGTGTACAA 3’. We used primers for human GM-CSF ori #2 (#23) [81]. The ‘exon 9’ primers amplify a region six kb upstream of the upper regulatory region of the MCM4 gene at which pre-ORC factors are not enriched [35]. Primer sets for the HPV-31 and HPV-16 LCR region in Fig 3 are listed in Table 1 below.

**Table 1:** HPV-31 and HPV-16 LCR primers used in Fig 3.

Primer Set	Sequence 5'-3'
HPV31L1_F	cacctccctcaggttctttg
HPV31L1_R	atggatcttccttgggcttt
HPV31LCR1_F	gcaccctcagcatctaccac
HPV31LCR1_R	cagcacaagcacatacacataca
HPV31LCR2_F	cctgctcctcccaatagtca
HPV31LCR2_R	ggaccgggtgtacaactttt
HPV31LCR3_F	gttctgcggtttttggtttc
HPV31LCR3_R	tgttggcaaggtgtgttagg
HPV31LCR4_F	aaagtggtgaaccgaaaacg
HPV31LCR4_R	catgcaatttccgaggtctt
HPV16L1_F	cagtttcctttaggacgcaaa
HPV16L1_R	gatgaggtggtgggtgtagc
HPV16LCR1_F	tgtttgtatgtgcttgtatgtgc
HPV16LCR1_R	gcatgacacaatagttacacaagc
HPV16LCR2_F	gccaaccattccattgtttt
HPV16LCR2_R	cgttggcgcatagtgattta
HPV16LCR3_F	aaactgcacatgggtgtgtg
HPV16LCR3_R	ttcggttacgcccttagtttt
HPV16E6_F	aatgtttcaggacccacagg
HPV16E6_R	ctcacgtcgcagtaactgttg

Results were analyzed using BioRad CFX manager software. Each experiment was performed at least three independent times.

### Flow cytometry

Cells were synchronized (G1/S) using 2.5 mM thymidine for 16 hr, released for 6 hr, and retreated with 2.5 mM thymidine for 16 hr before harvest. At harvest, cells placed in 90% ethanol. For processing, cells were washed in PBS, treated with 50 μg/ml RNase A, 0.05 mg/ml propidium iodide for 20 min, run on FACS Caliber and analyzed with FlowJo software.

### Co-immunoprecipitations and immunoblotting

Cells were transfected using Lipofectamine 2000 or PEI (2 mg/ml) according to manufacturers’ instructions. For experiments using MG-132, cells were treated 24 hr post transfection at 10 μM for 6 hr. After 24–48 hr, cells were lysed in 1% NP-40 containing 50 mM NaCl, 10 mM HEPES, pH 7.4, 10% glycerol, 2 μl benzonase (Millipore) and protease inhibitor cocktail (Sigma). To each reaction, 40 μl of 50% protein A/G slurry (Invitrogen) and 1 μg of antibody was added and rotated for 2 hr or overnight at 4°C. Beads were washed 2 times in PBS and 2 times in 500 mM NaCl, 0.1% SDS, 1% NP-40, 2 mM EDTA, 20 mM Tris-HCl, pH 8.0. Proteins were separated by SDS-polyacrylamide gel electrophoresis (SDS-PAGE). Gels were transferred onto PVDF membranes (Millipore), blocked in 5% PBS-Tween (0.1%) milk, and probed with antibodies. Chemiluminescence substrates (Thermo Scientific) were used to detect antibody signal.

Purification of 6X His E2 1–216 [82] and MBP-16E6 [83] proteins was performed as described [82, 84]. ORC2 baculovirus and ORC2 protein was produced as described elsewhere [85]. High Five cells infected with ORC2 baculovirus were lysed in 1% NP-40 buffer. Lysates were added to 6X His E2 1–216 and MBP-16E6 proteins with nickel Dynabeads (Invitrogen) beads in 1% NP-40 containing 50 mM NaCl, 10 mM HEPES, pH 7.4, 10% glycerol, 2 μl benzonase and protease inhibitor cocktail.

### Luciferase DNA replication assays

C33A cells were grown to 50% confluence in 6-well dishes. 3 μg shRNA plasmid (Control or ORC2 shRNA), 15 ng pRL (Rluc), 75 ng pFLORI31 or pFLORIBPV-1, 0.3 μg pE1 and 0.3 μg pE2 or 0.1 μg pREP4 (Life Technologies) using Lipofectamine 2000. For the BPV replication assays, pCG-BPV-1 E1 Eag1235 was used for E1 expression. 48–72 hr later cells were lysed and luciferase activity was measured using Steady-Glo or Dual Glo. Firefly luciferase levels were normalized to renilla luciferase levels. CIN612-9E cells were cultured to 50% confluence in 6-well dishes. Feeder J2 3T3 cells were removed before transfection. 0.5 μg shRNA plasmid (Control shRNA or ORC2 shRNA), 15 ng pRL (Rluc), 75 ng pFLORI31 were transfected into CIN612-9E cells using FugeneHD (Invitrogen). J2 3T3 cells were added the next day. 48 hr later, cells were lysed and luciferase activity measured using Dual Glo luciferase assay reagent (Promega). Firefly luciferase activity was normalized to renilla luciferase levels.

CIN612-9E cells, without feeders, were plated in 6 cm plates with the addition of the transfection reaction. The transfection reaction contained Lipofectamine 2000 (Invitrogen) and either control siRNA (Santa Cruz; sc-37007) or ORC2 siRNA duplexes (UUGAAGAAGGAGCGAGCGCAGCUUU [66], IDT) at a final concentration of 5 or 15 nM with 250 ng pFLORI31 and 50 ng pRL (Rluc) per well. Five hours post transfection, media containing the transfection was removed and replaced with E-medium or 10% FBS DMEM + 2 mM CaCl_2_ to induce differentiation. 48 hr later, cells were lysed and luciferase activity measured using Dual Glo luciferase assay reagent (Promega). Firefly luciferase activity was normalized to renilla luciferase levels.

### PCR DNA replication assays

HPV-BP and SiHa cells were plated in 10 cm plates. ShRNA knockdown hairpins were transfected using Fugene HD. 24 hrs post transfection cells were maintained in 1 μg/ml puromycin (Life Technologies) for 3–5 d. At about day 10, cells were lysed into Steady Glo (Promega) lysis buffer and western blot was performed as described above or cells were lysed in phenol:chloroform:isoamyl alcohol (25:24:1, Fisher Scientific). DNA was quantified using the Nanodrop and 30 ng was used for Real Time PCR as described above. HPV-16 DNA content was measured using primers to HPV-16 LCR (listed above-Fig 3). HPV-16 DNA content was normalized to a region of genomic DNA (GM-CSF ori # 2, listed above). This experiment was performed three independent times.

CIN612-9E cells, without feeders, were plated in 6 cm plates with the addition of the transfection reaction. The transfection reaction contained Lipofectamine 2000 (Invitrogen) and either control siRNA (Santa Cruz; sc-37007) or ORC2 siRNA duplexes (UUGAAGAAGGAGCGAGCGCAGCUUU [66], IDT) at a final concentration 15 nM. 4 hr post transfection, the media was replaced with 10% FBS DMEM + 2 mM CaCl_2_ to induce differentiation. 72 hr later, cells were lysed in TE with 0.1% SDS with 10 ng/μl RNase. DNA was isolated with phenol:chloroform:isoamyl alcohol (25:24:1, Fisher Scientific) and Real Time PCR was performed as described above. HPV-31 DNA content was measured using primers to HPV-31LCR3 (listed above-Fig 3). HPV-31 DNA content was normalized to a β-actin DNA primer set—5’ GAGGCACTCTTCCAGCCTTC 3’ and 5’ CGGATGTCCACGTCACACTT 3’.

### Immunofluorescence

CV-1 cells were transfected with 1 μg FLAG-tag HPV-31 E2, 1 μg FLAG-tag HPV-31 E1, and 8 μg YFP-ORC2 mammalian expression vectors with and without 50 ng HPV-31 origin or transfected with 2ug pCG-BPV-1 E2, 2ug pCG-BPV-1 E1, and FLAG mouse ORC2 mammalian expression vectors using Lipofectamine 2000. 24–48 hr later, coverslips were washed twice in PBS and fixed in 4% paraformaldehyde for 15 min followed by 3X PBS washes. The cells were permeabilized in PBS/0.2% Triton-X (PBS-T) for 10 min followed by 2X PBS washes. Coverslips were placed in 10% goat serum in PBS-T for 1 h, followed by primary antibody in PBS-T for 2 hr, washed 3X with PBS, incubated in 1:5000 dilution of goat anti-mouse 594 or goat-anti-rabbit 488 (Invitrogen) for 1 hr, washed 3X PBS, and mounted onto slides with Vectashield mounting media with DAPI.

### Ethics statement

All animal studies were carried out in accordance with the recommendations in the Guide for the Care and Use of Laboratory Animals of the National Institutes of Health and with the prior approval of the Animal Care and Use Committee of Johns Hopkins University under protocol RA14M47.

### Statistical analysis

Two-way or one-way t-test was used for analysis. Means are expressed +/- SEM. * indicates p-values ≤ 0.05.

## Supporting Information

S1 Fig ORC2 does not localize to HPV-31 E1-E2 replication foci. CV-1 cells were transfected with FLAG-HPV-31 E2, FLAG-HPV-31 E1, and YFP-ORC2 expression vectors with (A-C) or without 50 ng HPV-31 origin plasmid (D-F). Cells were fixed, permeabilized, and reacted with M2 antibody and nuclei were stained with DAPI (blue). E1 and E2 proteins are shown in red and YFP-ORC2 protein in green. Representative images for E1-E2-ORC2, E1-E2 alone, and ORC2 alone are shown.
(TIF)

S2 Fig ORC2 does not co-localize to BPV-1 E1-E2 replication foci. CV-1 cells were transfected with BPV-1 E2 and E1, and FLAG-mouse ORC2 expression vectors and reacted with B201 E2, rabbit E1, or rabbit FLAG or M2 antibodies. Cell nuclei were stained with DAPI (blue). Representative images are shown for E2 (A) and E1 (B) co-localization (C). E2 (D) and FLAG-ORC2 (E) show no co-localization (F). FLAG-ORC2 (G) with E1 (H) do not show co-localization (I).
(TIF)

S3 Fig ORC2 knockdown enhances PV replication. (A) ORC2 knockdown does not change luciferase levels. C33A cells transfected with control shRNA plasmid and pFLORI31 (ori) and luciferase levels were compared to groups containing ORC2 shRNA plasmid and pFLORI31 (ori) with either E1 or E2. Values are expressed as mean +/- SEM. (B) Replication luciferase assays were completed with pFLORIBPV-1. Values are expressed as mean +/- SEM. * p-value ≤ 0.05. (C) ORC2 shRNA enhanced HPV-31 replication in CIN612-9E cells at endogenous levels of E1 and E2. CIN612-9E cells transfected with 0.5 μg of shRNA, 15 ng RLuc and 75 ng pFLORI31 were lysed and luciferase activity measured. ORC2 shRNA decreased ORC2 protein levels in the CIN612 cells.
(TIF)

S4 Fig Cell Cycle Profiles. (A) Cell cycle profiles for 15 nM control and ORC2 siRNA in CIN612-9E cells at 48 h. (B) TRE-x U2OS cells containing pcDNA4/TO-FLAG 31E2 (i31E2, ± 48h Dox treatment) and Control and 16E2 were analyzed for cell cycle by flow cytometry.
(TIF)
